# paraFaceTest: an ensemble of regression tree-based facial features extraction for efficient facial paralysis classification

**DOI:** 10.1186/s12880-019-0330-8

**Published:** 2019-04-25

**Authors:** Jocelyn Barbosa, Woo-Keun Seo, Jaewoo Kang

**Affiliations:** 1 Department of Computer Science and Engineering, Korea University, Seoul, South Korea; 2 IT Department, University of Science and Technology of Southern Philippines, Cagayan de Oro, Philippines; 3 Department of Neurology and Stroke Center, Samsung Medical Center, Seoul, South Korea; 4 Sungkyunkwan University School of Medicine, Department of Digital Health, SAIHST, Sungkyunkwan University, Seoul, South Korea

**Keywords:** Facial paralysis classification, Facial paralysis objective evaluation, Ensemble of regression trees, Salient point detection, Iris detection, Facial paralysis evaluation framework

## Abstract

**Background:**

Facial paralysis (FP) is a neuromotor dysfunction that losses voluntary muscles movement in one side of the human face. As the face is the basic means of social interactions and emotional expressions among humans, individuals afflicted can often be introverted and may develop psychological distress, which can be even more severe than the physical disability. This paper addresses the problem of objective facial paralysis evaluation.

**Methods:**

We present a novel approach for objective facial paralysis evaluation and classification, which is crucial for deciding the medical treatment scheme. For FP classification, in particular, we proposed a method based on the ensemble of regression trees to efficiently extract facial salient points and detect iris or sclera boundaries. We also employ 2^*nd*^ degree polynomial of parabolic function to improve Daugman’s algorithm for detecting occluded iris boundaries, thereby allowing us to efficiently get the area of the iris. The symmetry score of each face is measured by calculating the ratio of both iris area and the distances between the key points in both sides of the face. We build a model by employing hybrid classifier that discriminates healthy from unhealthy subjects and performs FP classification.

**Results:**

Objective analysis was conducted to evaluate the performance of the proposed method. As we explore the effect of data augmentation using publicly available datasets of facial expressions, experiments reveal that the proposed approach demonstrates efficiency.

**Conclusions:**

Extraction of iris and facial salient points on images based on ensemble of regression trees along with our hybrid classifier (classification tree plus regularized logistic regression) provides a more improved way of addressing FP classification problem. It addresses the common limiting factor introduced in the previous works, i.e. having the greater sensitivity to subjects exposed to peculiar facial images, whereby improper identification of initial evolving curve for facial feature segmentation results to inaccurate facial feature extraction. Leveraging ensemble of regression trees provides accurate salient points extraction, which is crucial for revealing the significant difference between the healthy and the palsy side when performing different facial expressions.

## Background

Facial paralysis (FP) or facial nerve palsy is a neuromotor dysfunction that losses voluntary muscles movement in one side of the human face. As a result, this leads to the loss of the person’s ability to mimic facial expressions. FP is not an uncommon medical condition. For every sixty people around the world, one of them can be affected by facial paralysis [1]. Facial paralysis often causes patients to be introverted and eventually suffer from social and psychological distress, which can be even more severe than the physical disability [2]. It is usually encountered in clinical practices, which can be classified into peripheral and central facial palsy [3, 4]. These two categories differ from each other according to the behavior of the upper layer of the human face. Peripheral facial palsy is a nerve disturbance in the pons of the brainstem, which affects the upper, middle and lower facial muscles of one side of the face. On the other hand, central facial palsy is a nerve dysfunction in the cortical areas whereby the forehead and eyes are spared, but the lower half of one side of the face is affected, unlike in peripheral FP [3–5]. Such scenario has triggered the interest of researchers and clinicians of this field, and, consequently, led them to the development of objective grading facial functions and methods in monitoring the effect of medical, rehabilitation or surgical treatment.

Many computer-aided analysis systems have been introduced to measure dysfunction of one part of the face and the level of severity, but not much on facial paralysis type as the classification problem. Moreover, the efficiency of the method used for it to be universally accepted is still underway [3]. Classification of each case of facial paralysis into central or peripheral plays a critical role in helping physicians to decide for the most appropriate treatment scheme to use [4]. Image processing has been applied in the existing objective facial paralysis assessment, but the processing methods used are mostly labor-intensive, if not; suffer from the sensitivity to the extrinsic facial asymmetry caused by orientation, illumination and shadows. As such, creating a clinically usable and reliable method is still very challenging. Wachtman et al. [6, 7] measured facial paralysis by examining the facial asymmetry on static images. Their methods are prone to facial asymmetry even for healthy subjects due to sensitivity to orientation, illumination and shadows [8]. Other previous works [9–11] were also introduced but most of them are solely based on finding salient points on human face with the use of the standard edge detection tool (e.g. Canny, Sobel, SUSAN) for image segmentation.

Canny edge detection algorithm may yield to inaccurate results as it influences connected edge points. This is because it does comparisons of the adjacent pixels on the gradient direction to determine if the current pixel has a local maximum. Improper segmentation will also result to improper generation of key points. Moreover, it may be difficult to find and detect the exact points when the same algorithm is applied to elder patients. Dong et al. [11] proposed the use of salient points for degree of facial paralysis estimation. A method was proposed to detect the salient points that will be the basis for the estimation of the degree of paralysis. As the salient points may include some unnecessary points for describing facial features, edge detection was used to discard these points. Then K-means clustering is applied to classify these salient points into six categories: 2 eyebrows, 2 eyes, nose, and mouth. There are about 14 key points are found in the six facial regions, which would represent the points that may be affected when performing some facial expressions. However, this technique falls short when applied to elder patients, in which exact points can be difficult to apply [12].

Another method was proposed that estimates the degree of facial paralysis by comparing multiple regions on human face. In [13], Liu et al. perform comparison of the two sides of the face and compute the four ratios, which are consequently used to represent the severity of the paralysis. Nevertheless, this method suffers from the influence of uneven illumination [8].

**Fig. 1:**
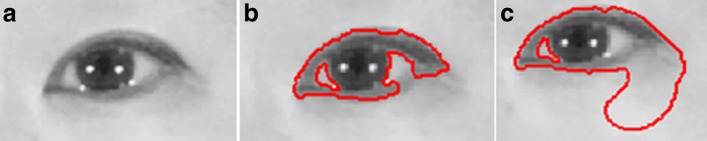
Pre-processing results. **a** eye image with some uneven illumination, **b**-**c** extracted eye contour by LAC model when parameters of initial evolving curves are not properly identified

In our previous work [4], a technique that generates closed contours for separating outer boundaries of an object from background using Localized Active Contour (LAC) model for feature extraction was employed, which reasonably reduced these drawbacks. However, one limiting factor of the method introduced is that it has a greater sensitivity to subjects exposed to peculiar images where facial features suffer from different occlusions (e.g. eyeglasses, eyebrows occluded with hair, wrinkles, excessive beard, etc.). Moreover, improper setting or identification of parameters, such as the radius of the initial evolving curve (e.g. minimum-bounding box of the eyes, eyebrows, etc.) may lead to improper features segmentation, which may in turn gives inaccurate detection of key points as revealed in Fig. 1. Although such limitation was addressed in [4] by applying a window kernel (i.e. generated based on Otsu’s method [14]) that is run through the binary image (e.g. detected eyes, eyebrows, lip, etc.), Otsu’s method has some drawbacks of the assumption of uniform illumination. Additionally, it does not use any object structure or spatial coherence, which may sometimes result to inaccurate generation of kernel paramaters that may in turn result to improper segmentation.

In order to address these drawbacks, we present a novel approach based on ensemble of regression trees (ERT) model. We leverage ERT model to extract salient points as basis for preparing the features or independent variables. Our target was to keep a natural framework that finds 68 key points on edges of facial features (e.g. eyes, eyebrows, mouth, etc.); and regresses the location of the facial landmarks from a sparse subset of intensity values extracted from an input image. As ERT model provides a simple and intuitive way in performing this task and in generating landmarks that separates boundaries of each significant facial feature from the background, without requiring setting-up of parameters for initial evolving curves as required by the previous approach [4], we find this technique appropriate to address our problem. Furthermore, empirical results [15] reveal that ERT model does have an appealing quality that performs shape invariant feature selection while minimizing the same loss function during training and test time, which significantly lessens time complexity of extracting features.

In this study, we make three significant contributions. First, we introduce a more robust approach for efficient evaluation of facial paralysis and classification, which is crucial for determining the appropriate medical treatment scheme. Second, we provide an efficient method in feature extraction based on ensemble of regression trees in a computationally efficient way; and finally, we study in depth, the effect of computing the facial landmarks symmetry features on the quality of facial paralysis classifications.

## Methods

The study performs objective evaluation of facial paralysis particularly the facial classification and grading in a computationally efficient way. We capture facial images (i.e. still photos) of the patients with a front view face and with reasonable illumination of lights so that each side of the face achieves roughly similar amount of lighting. The photo taking procedure starts with the patient performing the ’at rest’ face position, followed by the three voluntary facial movements that include raising of eyebrows, screwing-up of nose, and showing of teeth or smiling.

**Fig. 2:**
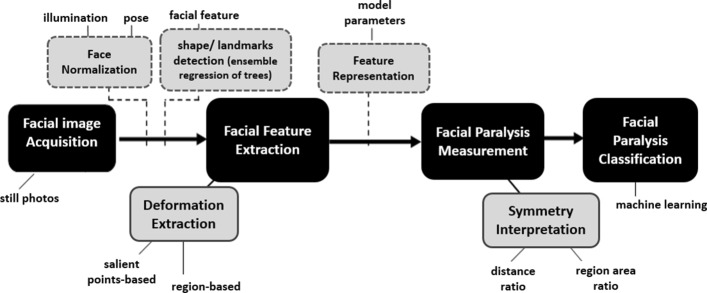
Framework of the proposed objective facial paralysis evaluation

The framework of the proposed objective facial paralysis evaluation system is presented in Fig. 2. Facial images of a patient, which are taken while being requested to perform some facial expressions, are stored in the image database. In the rest of this section, we describe the details of the form of individual components of the facial landmark detection and how we perform evaluation and classification of facial paralysis. To begin the process, raw images from the image database are extracted or retrieved. Dimension alignment and image resizing are then performed, followed by the pre-processing of images to enhance contrast and remove undesirable image noise.

### Facial Image Acquisition and Pre-processing

Existing literatures show that facial data acquisition methods can be classified into two categories depending on the processing methods they used and on the usage of images or videos as their databases. As image-based systems do have an appealing advantage of ease of use and cost effectiveness [16], we utilize still images as inputs to our classification model.

#### Face Normalization

Facial feature extractions can be complicated as the face appearance changes, which is caused by illumination and pose variations. Normalizing the acquired facial images prior to objective FP assessment may significantly reduce these complications. It is worth note taking, however, that while face normalization may be a good approach in connection with the methods for objective facial paralysis assessment, it is but optional, so long as extracted feature parameters are normalized prior to the classification task.

### Facial Feature Extraction

#### Deformation Extraction

Facial features deformation can be categorized by the changes of texture and shape that lead to high spatial gradients, which are good indicators for tracking facial actions [17]. In turn, these are good indicators for facial asymmetry and may be analyzed either in image or spatial frequency domain, which can be computed by high-pass gradient or Gabor wavelet-based filters. Ngo et al. [18] utilized this method by combining it with local binary patterns and claimed to perform well for the task of quantitative assessment of facial paralysis. Deformation extraction approaches can be holistic or local. Holistic image-based approaches are those where face is processed as a whole, while local methods focused on facial features or areas that are proned to change and are able to detect subtle changes in small areas [17]. In this work, we extract the facial salient points and iris area for geometric-based and region-based features generation, respectively.

#### Salient points detection

Feature extraction starts from the preprocessing of the input image and facial region detection. We find frontal human faces in an image and estimate their pose. Such estimated pose takes the facial form of 68 landmarks that will lead to the detection of the key points of the mouth, eyebrows and eyes. The face detector is made using the classic Histogram of Oriented Gradients (HOG) feature [19] combined with a linear classifier, an image pyramid, and sliding window detection scheme. We utilize the pose estimator employed in [15]. In the context of computer vision, a sliding window is a rectangular region of fixed width and height that slides across an image, as described in the subsequent section. Normally, for each window, we take the window region and apply an image classifier to check if the window has an object (i.e. the face as our region of interest). Combined with image pyramids, image classifiers can be created that can recognize objects even if the scales and locations in the image vary. These techniques, in its simplicity, play an absolutely critical role in object detection and image classification.

**Fig. 3:**
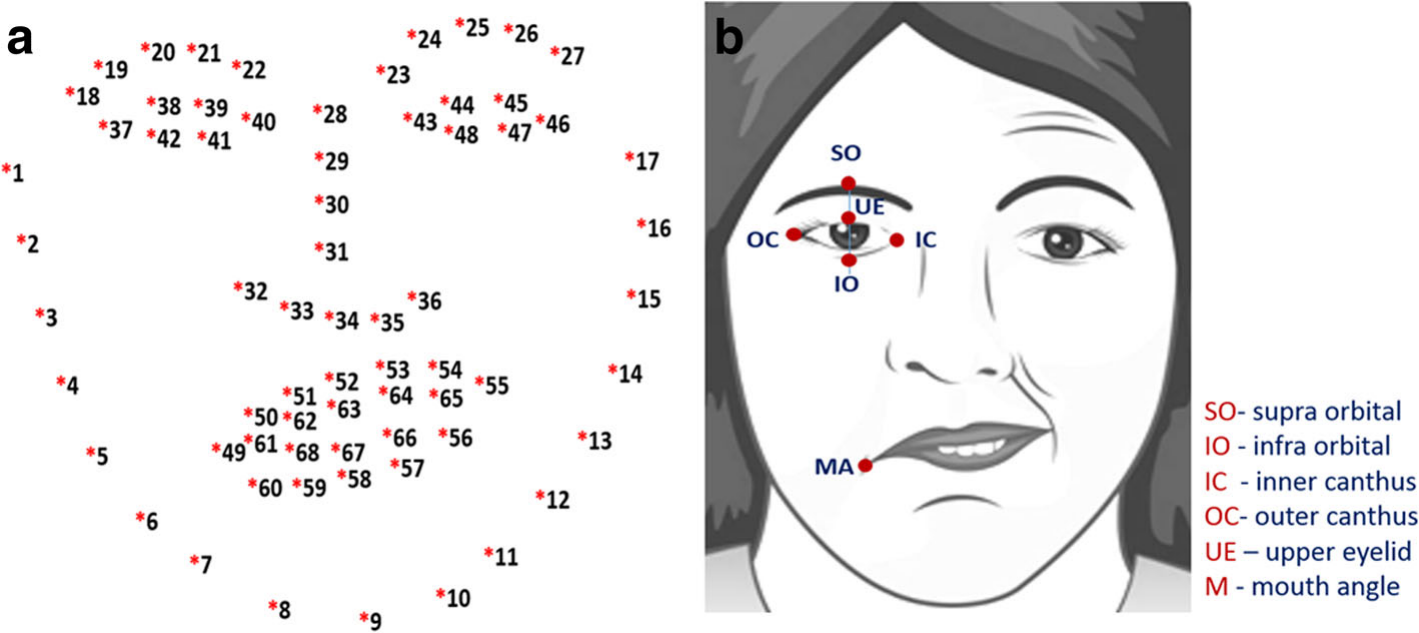
Facial landmarks or key points. **a** 68 key points, and **b** salient points for each facial feature utilized in this study

**Fig. 4:**
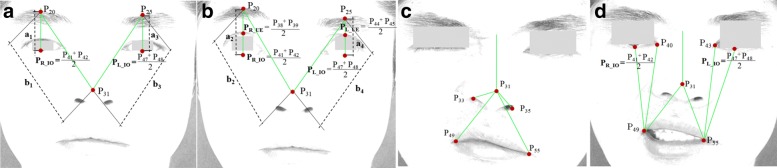
Facial expressions used in the study. **a** at rest; **b** raising or lifting of eyebrows; **c** screwing-up of nose; and **d** smiling or showing of teeth

Features are extracted from the different facial expressions that the patient will perform, which include: (a) at Rest; (b) Raising of eyebrows; (c) frowning or screwing up of nose; and (d) Smiling or showing of teeth. In this study, we consider geometric and region-based features as our inputs for modelling a classifier. To extract the geometric-based features, the salient points of the facial features (e.g. eyes, eyebrows and lip) are detected using the concept of ensemble of regression trees [15]. The goal is to find the 68 key points on edges of facial features as shown in Fig. 3a. However, for generating the geometric-based symmetry features of each image, we are interested in the following points: inner canthus (IC), mouth angle (MA), infra orbital (IO), upper eyelids (UE), supra-orbital (SO), nose tip (NT) and nostrils (see Fig. 3b). In order to detect the salient points of the facial image, we leverage the dlib library [15] that has the capability to detect points of each facial features. These points are inputs for calculating the distance of the key points as identified in Fig. 4, and the corresponding distance ratio for both sides of the face. Additionally, we also extract region-based features, which involves detection of iris or sclera boundaries by leveraging Daugman’s Integro-Differential Operation [20].

#### Histogram of Oriented Gradients(HOG)

In the field of computer vision and image processing, Histogram of Oriented Gradients (HOG) is a feature descriptor used for object or face detection. It is an algorithm, which takes an image and outputs feature vectors or feature descriptors. Feature descriptors encode interesting information into a series of numbers and act as a sort of numerical fingerprint, which can differentiate one feature from another. Ideally, this information is invariant under image transformation; hence, even if the image is transformed in some way, we can still find the feature again. HOG uses locally normalized histogram of gradient orientation features similar to Scale Invariant Feature Transform (SIFT) descriptor in a dense overlapping grid, which gives very good results in face detection. It is similar to SIFT, except that HOG feature descriptors are computed on a dense grid of uniformly spaced cells and they use overlapping local contrast normalization for improved performance [19, 21].

Implementation of the HOG descriptor algorithm is as follows:

1. Partition the image into small connected regions called cells, then for each cell, calculate the gradient directions histogram for the pixels within the cell.
2. According to the gradient orientation, discretize each cell into angular bins. Each pixel of the cell contributes weighted gradient to its corresponding angular bin. Adjacent group of cells are considered as spatial regions, referred to as blocks. The grouping of cells into a block is then the basis for histograms grouping and normalization. Normalized group of histograms represents the block histogram. The set of these block histograms represents the descriptor.

The following basic configuration parameters are required for computation of the HOG descriptor:

1. Masks to compute derivatives and gradients.
2. Geometry of splitting an image into cells and grouping cells into a block.
3. Block overlapping
4. Normalizing parameters

However, the recommended values for the HOG parameters include: (a)1D centered derivative mask [-1, 0, +1]; (b) Detection window size of 64x128; (c) Cell size of 8x8; and Block size of 16x16 (2x2 cells) [21].

#### Ensemble of Regression Trees

An ensemble of regression tree is a predictive model, which composes a weighted combination of multiple regression trees. Generally, combining multiple regression trees increases the predictive performance. It is with the collection of regression trees that makes a bigger and better regression tree [15]. The core of each regression function *r*_*t*_ is the tree-based regressors fit to the residual targets during the gradient boosting algorithm.

#### Shape invariant split tests

Based on the approach used by Kazemi et al. [15], we make a decision based on thresholding the difference between the intensities of two pixels at each split node in the regression tree. When defined in the coordinate system of the mean shape, the pixels used in the test are at positions *i* and *k*. For facial images having arbitrary shapes, we intend to index the points that have the same position relative to its shape, as *i* and *k* have to the mean shape. To accomplish this task, the image can be warped to the mean shape based on the current shape estimate before extracting the features. Warping the location of points is more efficient rather than the whole image, since we only utilize a very sparse representation of the image. In what follows, the details are precisely shown. We let *vi* be the index of the facial landmark in the mean shape that is nearest to *i* and its offset from *i* is defined as:

1 $$  \delta {Y_{i}} = i - {\overline Y_{vi}}\  $$

Then for a shape *S*_*j*_ defined in image *I*_*j*_, the position in *I*_*j*_ that is qualitatively similar to *i* in the mean shape image is given by

2 $$  i^{\prime} = {Y_{j,vi}} + \frac{1}{{{s_{j}}}}R_{j}^{\mathrm{T}}\delta {Y_{i}}\  $$

where *S*_*j*_ and *R*_*j*_ are the scale and rotation matrix of the similarity transform which transforms *S*_*j*_ to $\overline {S}$, the mean shape. The scale and rotation are found to minimize

3 $$ \sum\limits_{m = 1}^{n} {||} {\overline{Y}}_{m} - \left({s_{j} R_{j} Y_{j,m} + t_{j}} \right) {||}^{2} \  $$

the sum of squares between the mean shape’s facial landmark points, $\overline {Y}_{m}$’s, and those of the warped shape. *k*^′^ is similarly defined. Formally each split is a decision involving 3 parameters *θ*=(*τ*,*i*,*k*) and is applied to each training and test example as

4 $$  h\left({I_{\pi_{j}},{\widehat{S}}_{j}^{(t)},\theta} \right) = \left\{\begin{array}{ll} {1} & {I_{\pi_{j}} \left({i^{\prime}} \right) - I_{\pi_{j}} \left({k^{\prime}} \right) > \tau }\\ {0} & {Otherwise} \end{array}\right.  $$

where *i*^′^ and *k*^′^ are defined using the scale and rotation matrix which best warp $\hat {S}^{(t)}_{j}$ to $\overline {S}$ according to equation (2).

In practice, during the training phase, the assignments and local translations are identified. Computing the similarity transform, at test time, the most computationally expensive part of the process is only done once at every level of the cascade.

This method starts by using the following:

1. A training set of labeled facial landmarks on an image. These images are manually labeled, specifying specific (x, y)-coordinates of regions surrounding each facial structure.
2. Priors, or more specifically, the probability on distance between pairs of input pixels.

**Fig. 5:**
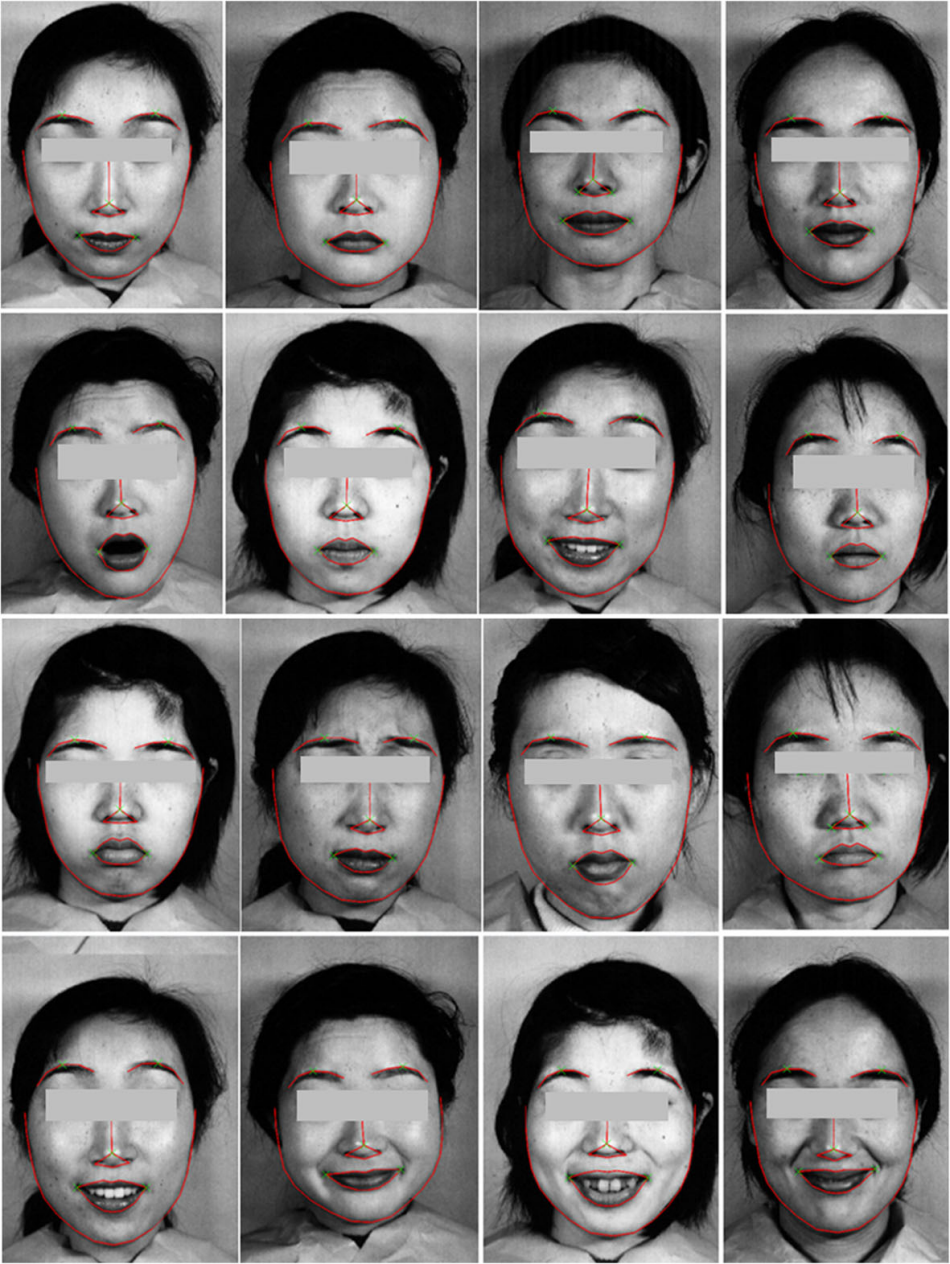
Sample results of our proposed approach for facial feature extraction based on Ensemble of Regression Trees (ERT) model as applied in JAFFE dataset [22]. Facial landmarks (i.e. 68 key points) are detected from a single image, based on ERT algorithm

Given this training data, ensemble of regression trees are trained to estimate the positions or locations of facial landmarks directly from the pixel intensities, that is, no feature extraction is taking place. The final result is a detector of facial landmarks that can be utilized to efficiently detect salient points of an image. Fig. 5 presents sample results of our proposed approach for facial features detection based on ensemble of regression trees (ERT) model when applied in JAFFE dataset [22, 23].

#### Region-based Feature Extraction

#### Iris Detection

A person who has a symptom of facial paralysis is likely to have asymmetric distance between the infra orbital (IO) and mouth angle (MA) of both sides of his face while performing facial movements such as smile and frown. They may also have an unbalanced exposure of iris when performing different voluntary muscle movements (e.g. screwing of nose, showing of teeth or smiling, raising of eyebrows with both eyes directed upward) [4]. We utilize the points (e.g. upper eyelid (UE), infra orbital (IO), inner canthus (IC) and outer canthus (OC)) detected by ensemble of regression trees algorithm and from there, we generate the parameters of the eye region as inputs to Daugman’s Integro Differential operator [20] to detect the iris sclera or boundaries. However, as some eye images do have eyelid occlusions, optimization of Daugman’s algorithm was performed to ensure proper iris boundaries detection. In our previous work, LAC-based method was employed to optimize Daugman’s algorithm and perform subtraction method. However, LAC model is quite tedious as it may result to improper segmentation if the initial evolving curves and iterations are not properly defined. Moreover, it has a greater sensitivity to the eyes of old patients due to the presence of excessive wrinkles.

In this paper, we implement the curve fitting scheme to ensure proper detection of iris sclera or boundaries, thereby providing better results in detecting occluded iris. By definition, a parabola is a plane curve. It is said to be mirror-symmetrical and is approximately U-shaped. To fit a parabolic profile through the data, a second degree polynomial is used, defined as: *y*=*ax*^2^+*bx*+*c*

This will exactly fit a simple curve to the three constraints, which include a point, angle, or curvature. More often, the angle and curvature constraints are added to the end of a curve. In this case, they are called end conditions. To fit a curve through a data, we first get the coefficients *b* and *c* of the line p(x) = bx + c. To evaluate this line, values of x (e.g. given by xp) must be chosen. For example, the curve will be plotted for x belongs to [0, 7] in steps of delta x = 0.1. The generated coefficients will then be used to generate the y values of the polynomial fit at the desired values of x given by xp. This means that the vector (denoted as yp) can be generated, which contains the parabolic fit to the data evaluated at the x-values xp.

In what follows, we describe our approach for detecting the iris sclera or boundaries:

1. Detect eye region (i.e. creating a rectangular bounding box from the detected UE,IO, IC, OC as illustrated in Figure 3b)
2. Detect upper eyelid edge using gray thresh level (e.g. threshold <= 0.9)
3. Convert generated grayscale image to binary.
4. Find significant points of the upper eyelid.
  Traverse each column and find the first row whose pixel value=1 and whose location variance (i.e. row address of the last 4 pixels) is minimal within the threshold. We call it vector A.
5. Implement curve fitting through the generated data points using parabolic profile (i.e. second degree of polynomial). We refer this as A’.
6. Detect iris using Daugman’s integro-differential operator and convert it to binary form. We call it vector B.
7. Find intersections of the two vectors A’ and B and take all pixels of vector B below the parabolic curve A’.
8. Utilize the points of the lower eyelid detected ERT model, we call it vector C.
9. Finally, we find the the region of the detected iris within the intersection of vector B and C.

**Fig. 6:**
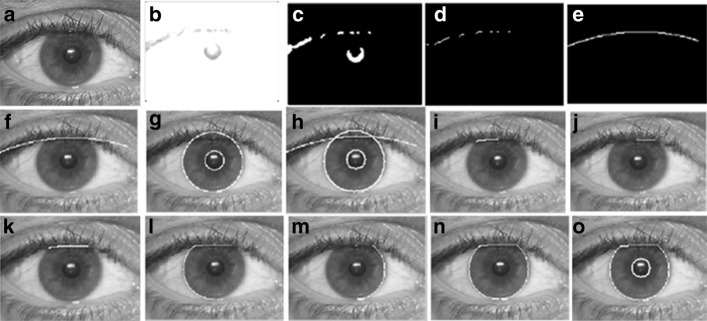
Extracted iris using our proposed approach based on Daugman’s algorithm. (**a**) converted gray scale image; (**b**) upper edge of the eye with gray thresh level of 0.9; (**c**) equivalent binary form of the detected upper eyelid; (**d**) data points of the upper eyelid; (**e**)-(**f**) upper eyelid as a result of employing parabolic function (2^*nd*^ degree polynomial); (**g**) result of Daugman’s Integro-Differential operator iris detection; (**h**)-(**n**) eyelid detection results using our optimized Daugman’s algorithm; and (**o**) final results of detecting iris boundaries with superior or upper eyelid occlusion

**Fig. 7:**
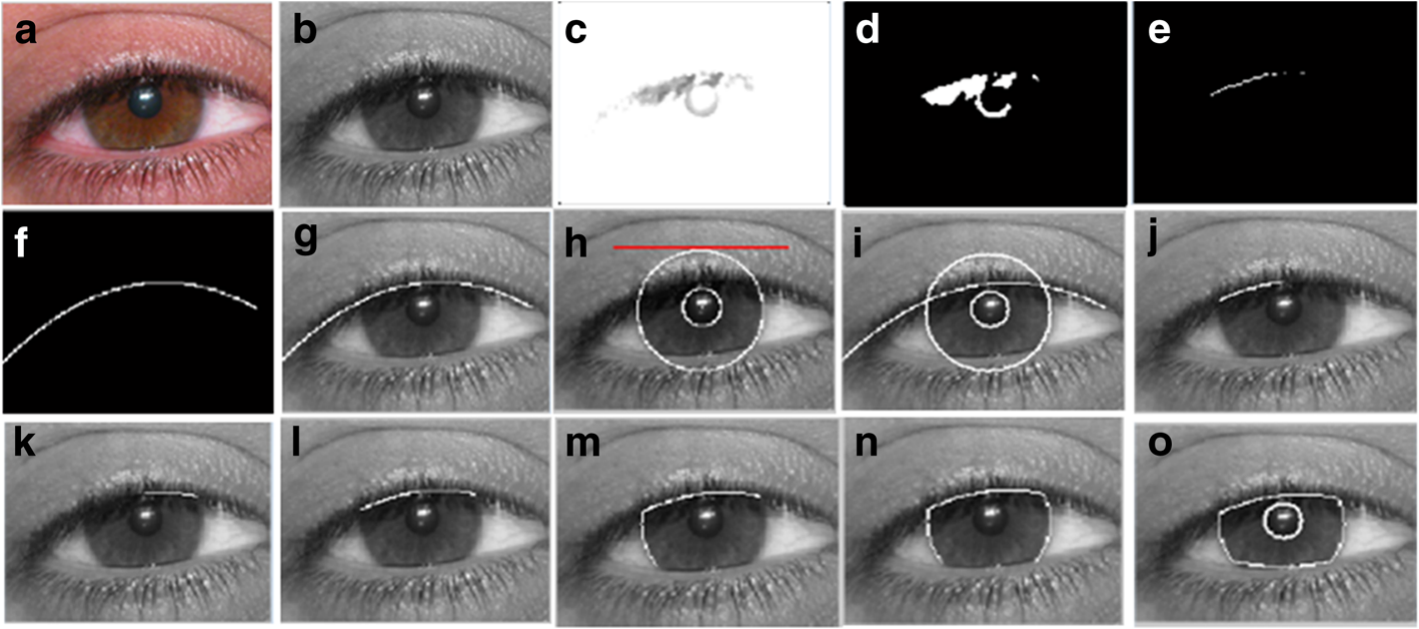
Some more results of extracted iris from the UBIRIS images [24]. **a** Original image; **b** Converted gray scale; **c** upper edge of the eye with gray thresh level of 0.9; **d** equivalent binary form of the detected upper eyelid; **e** data points of the upper eyelid; **f**-**g** upper eyelid as a result of employing parabolic function (2^*nd*^ degree polynomial); **h** result of Daugman’s Integro-Differential operator iris detection; **i**-**n** results eyelids detection with our optimized Daugman’s algorithm; and **o** final results of segmented iris occluded by upper and lower eyelids

A closer look at Figs. 6 and 7 reveal interesting results of this approach.

### Facial Paralysis Measurement

#### Symmetry Measurement by Iris and Key points

In this paper, the symmetry of both sides of the face is measured using the ratio that are obtained from computing the iris area (i.e. generated while the subject performs raising of eyebrows while both eyes are directed upward; and screwing of nose or frown) and the distance between the two identified points on each side of the face while the subject is asked to perform the different facial expressions (e.g. at rest, raising of eyebrows, screwing of nose, and showing of teeth or smile). Table 1 shows the summary of the salient points as basis for extracting features such as the ratio of iris area as well as the distance ratio between two sides of the face.

**Table 1:** List of facial expressions and the corresponding landmarks used for feature extraction

Facial expression	SO	IO	IC	MA	NT	N
at Rest	✓	✓				
	✓				✓	
Raising of eyebrows	✓	✓				
	✓				✓	
Smile			✓	✓		
		✓		✓		
				✓	✓	
Frown				✓	✓	
					✓	✓

With ’at rest’ and ’raising of eyebrows’, we calculate the distance between two points: Infra Orbital (IO) and supra-orbital (SO); and the Nose Tip (NT) and SO, while with ’smile’ expression, we get the distances between the two identified points: inner canthus (IC) and Mouth Angle (MA); IO and MA; and the NT and MA. Lastly, for ’frown’ expression, we get the distance between the two points: NT and MA; and NT and nostrils. Consequently, the computed distance ratio of both sides of the face are considered as the symmetrical features of each subject.

Computed distances include: *P*_20_*P*_R_IO_, *P*_25_*P*_L_IO_, *P*_20_*P*_31_ and *P*_25_*P*_31_ (see Fig. 4a and 4b); *P*_31_*P*_33_, *P*_31_*P*_35_, *P*_31_*P*_49_ and *P*_31_*P*_55_ (see Figure 4c); and *P*_40_*P*_49_, *P*_43_*P*_55_, *P*_31_*P*_49_, *P*_31_*P*_55_, *P*_49_*P*_R_IO_ and *P*_55_*P*_L_IO_ (see Figure 4d). Additionally, we calculate the area of the extracted iris. This is followed by the computation of ratio between two sides. We find the expression below:

5 $$ dRatio = \left\{\begin{array}{l} {\frac{D_{L}}{D_{R}} D_{R} > D_{L}}\\ {\frac{D_{R}}{D_{L}}otherwise} \end{array}\right.  $$

where *dRatio* is the ratio of the computed distance *D*_*L*_ and *D*_*R*_ of the specified key points of each half of the face.

We also consider the capability of the patients to raise the eyebrows (i.e. rate of movement) as one important features for symmetry measurement by comparing the two facial images, the ‘at rest position’ and ‘raising of eyebrows’ as shown in Fig. 4a and 4b. We compute the distances *a*_1_ and *b*_1_ (Fig. 4a) where *a*_1_ and *b*_1_ are the distance from *P*_20_ and $P_{\texttt {R\_IO}}$ and *P*_20_ and *P*_31_ of the right eye, respectively. We then compute the ratio of *a*_1_ and *b*_1_. Similarly, for the second image (Fig. 4b), we get *a*_2_ and *b*_2_ as well as the ratio. Finally, we compute the difference of these two ratio (i.e. difference between *a*_1_/ *b*_1_ and *a*_2_/ *b*_2_) and denote it as *right*_*Movement*.

The same procedure is applied to the two images for finding the ratio difference for the left eye (i.e. difference between *y*_3_/ *x*_3_ and *y*_4_/ *x*_4_) and denote it as *left*_*Movement*. The rate of movement can be computed by finding the ratio between *right*_*Movement* and *left*_*Movement*. Intuitively, the difference of these two ratio values for normal subjects is likely to be higher (usually approaching to 1, which signifies the ability to raise both of his eyebrows) than those of the FP patients.

### Facial Palsy Type Classification

Classification of facial paralysis type involves two phases: (1) discrimination of healthy from unhealthy subjects; and (2) proper facial palsy classification. In this context, we model the mapping of symmetry features (as described in the previous subsection) into each phase as binomial classification problem. As such, we employ two classifiers to be trained, one for healthy and unhealthy discrimination (0-healthy, 1-unhealthy) and another one for facial palsy type classification (0-peripheral palsy(PP), 1-central palsy(CP)). For each classifier, we consider Random Forest (RF), Regularized Logistic Regression (RLR), Support Vector Machine (SVM), Decision Tree (DT), naïve bayes (NB) and Hybrid classifier as appropriate classification methods as they have been successfully applied to pattern recognition and classification on datasets with realistic size [4, 8, 13, 22]. With hybrid classifier, we apply rule-based approach prior to employing machine learning (ML) method. This is based on empirical results [4, 11, 13], which show that normal subjects are likely to have an average facial measurement ratio closer to 1.0 and central palsy patients are likely to have a distance ratio from Supra-orbital (SO) to Infra-orbital (IO) approaching to 1.0. Similarly, iris exposure ratio usually results to values nearly close to 1. Hence, we find hybrid classifier (rule-based + ML) appropriate in our work.

**Fig. 8:**
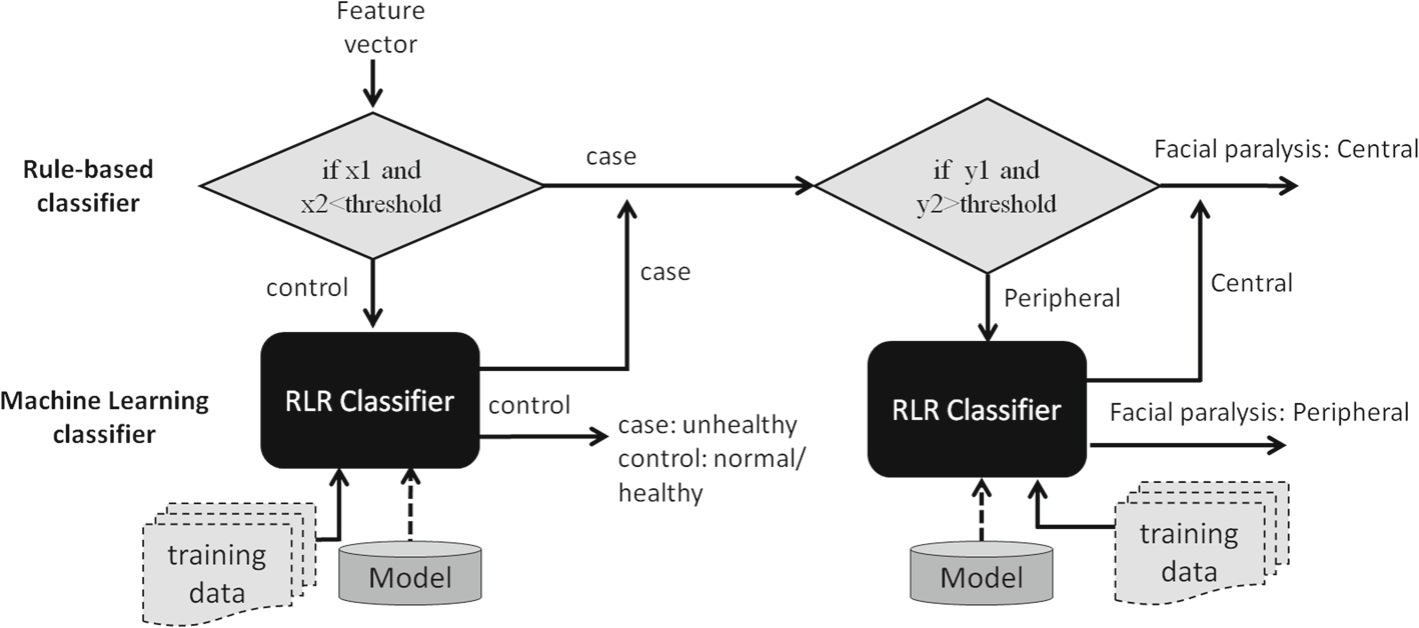
Hybrid Classifier

This process is presented in Fig. 8. If rule number 1 is satisfied, the algorithm continues to move to the case path (i.e. for the second task), making a test if rule number 2 is also satisfied; otherwise, it performs a machine learning task, such as RF, RLR, SVM, and NB. The rules are generated after fitting the training set to the DT model. For example, rule 1 may have conditions, like if *f*_*x*_<0.95and*f*_*y*_<0.95 (where *f*_*x*_and*f*_*y*_ are two of the predictors, the mean result of all parameters and the IO_MA ratio, respectively, based on Table 1), then the subject is most likely to be diagnosed with facial paralysis, and therefore can proceed for rule no. 2 (i.e. to predict the FP type); otherwise, it performs a machine learning task. If the classifier returns 0, the algorithm exits from the entire process as this signifies that the subject is classified as normal/healthy, else it moves to the case path to perform a test on rule number 2 for facial palsy type classification.

If rule number 2 is satisfied, the system gives 1 (i.e. 0-PP; 1-CP), else the feature set is fed to another classifier, which can yield either 0 or 1. The same with rule number 1, rule number 2 is also generated by DT model. For example, rule 2 may set conditions like, if *f*_*a*_>0.95 and *f*_*b*_>0.95 (where *f*_*a*_ and *f*_*b*_ are two of the predictors *SO*_*IO* ratio and iris area ratio, respectively), then it is most likely to be diagnosed as having central palsy (CP), otherwise, the feature set is fed to another classifier, which could return either 0 or 1 (i.e. 0-PP; 1-CP).

## Results

In our experiments, we used 440 facial images, which were taken from 110 subjects (i.e. 50 patients and 60 healthy subjects). From the 50 unhealthy subjects, 40 of which have peripheral palsy (PP) cases and 10 have central palsy (CP) cases. We used 70% of the dataset as the training set and 30% as the test set. For example, in discriminating healthy from unhealthy subjects, we used 77 subjects (i.e. 35 patients plus 42 normal subjects) as the training set and 33 subjects (i.e. 15 patients plus 18 normal) as the test set. While in FP type classification problem 35 unhealthy cases (i.e. 28 PP and 7 CP) as our training set and 15 (i.e. 12 PP and 3 CP) as our test set.

Each subject was asked to perform 4 facial movements. During image pre-processing, resolutions were converted to 960 x 720 pixels. Facial palsy type of each subject was pre-labeled based on the clinicians’ evaluation, which was used during the training stage. This was followed by the feature extraction or the calculation of the area of the extracted iris and the distances between the identified points as presented in Fig. 4. Overall, we utilize 11 features to train the classifier. The samples for healthy and unhealthy classifier were categorized into two labels: 0-healthy, 1-unhealthy. Similarly, samples for unhealthy subjects were classified into two labels: 0-central palsy and 1-peripheral palsy. It can be noted that healthy subjects have very minimal asymmetry in both sides of the face resulting to a ratio that approaches to 1.

### Facial paralysis type classification

Regularized logistic regression (RLR), Support Vector Machine (SVM), random forest (RF), naïve bayes (NB), and classification tree (DT) were also utilized to compare with our hybrid classifiers. Given the dataset that is not very large, we adopt the k-fold cross-validation test scheme in forming a hybrid model. The procedure involves 2 steps: rule extraction and hybrid model formation, as applied in our previous work [4].

#### Step 1: rule extraction.

If we have a dataset D = ((*x*_1_, *y*_1_),..., (*x*_*n*_, *y*_*n*_)), we hold out 30% of D and use it as a test set T = ((*x*_1_, *y*_1_),..., (*x*_*t*_, *y*_*t*_)), leaving 70% as our new dataset D’. We adopt k-fold cross-validation test scheme over D’, i.e. with k = 10. For example, if N = 80 samples, each fold has 8 samples. In each iteration, we leave one fold out and consider it as our validation set and utilize the remaining 9 folds as our training set to learn a model (e.g. rules extraction). Since we have 10 folds, we do this process for 10 repetitions. We extract rules by fitting the training set to a DT model.

#### Step 2: hybrid model formation.

In this step, we form a hybrid model by combining the rules generated in each fold and the ML classifier. Each model is tested out over the validation set using different parameters (e.g. lambda for logistic regression and gamma for SVM). For example, to form the first hybrid model, we combine the rule extracted from the first fold and a regularized logistic regression model (i.e. rule + RLR) and test out its performance over the validation set (left-out fold) while applying it to each of the 10 parameters. Therefore, for each fold, we generate 10 performance measures. We repeat this procedure for the succeeding folds, which means performing the steps for k times (i.e. with k = 10, since we are using 10-fold cross validation) will give us 100 performance measures. We calculate the average performance in all folds. This yields 10 average performance measures (i.e. for each parameter n), each of which corresponds to one specific hybrid model. Then we choose the best hybrid model, i.e. a model with lambda that minimizes errors. We retrain the selected model on all of D’, test this out over the hidden test set T = ((*x*_1_,*y*_1_),·,(*x*_*t*_,*y*_*t*_)), i.e. 30% of the dataset D and get the performance of the hybrid model.

We evaluate the classifiers by their average performance for 20 repetitions of k-fold cross-validation using k = 10. We repeat this process for evaluation of other hybrid model (e.g. rule-based + RF, rule-based + RLR, etc.) and finally choose the hybrid model that performs best. The hybrid classifiers, RF, SVM, RLR, DT and NB were tested and experiments reveal that hybrid classifier rule-based + RLR (hDT_RLR) outperformed other classifiers for discriminating healthy from unhealthy (i.e. with paralysis) subjects. Similarly, for the classification task of facial palsy (PP-peripheral and CP-central palsy), hDT_RLR hybrid classifier is superior among other classifiers used in the experiments.

Table 2 presents a comparison of the average performance of our hybrid classifier, RF, RLR, SVM, DT and NB based on our proposed approach. For FP type classification, our hDT_RLR hybrid classifier achieves a better performance on sensitivity of 5.2% higher than RLR, RF, SVM, DT and NB (see Table 2). Other hybrid classifiers also show good results comparable with hDT_RLR. However, in the context of our study, we are more concerned on designing a classifier that yields a stable results on sensitivity performance measure without necessarily sacrificing the specificity or fall-out or the probability of false alarm. Hence, we employ hDT_RLR.

**Table 2:** Comparison of the performance of different classifiers for facial palsy classification

classifier	Sensitivity(%)	Specificity(%)
RLR	85.9	97.7
RF	92.3	95.0
SVM	72.5	94.8
DT	90.2	94.0
NB	79.9	95.4
hDT_RLR	97.5	94.9
hDT_RF	94.3	95.4
hDT_SVM	96.9	90.0
hDT_NB	96.9	90.9
hLR_RF	92.2	94.1
hLR_SVM	85.9	94.8
hLR_DT	92.5	93.7
hLR_NB	85.9	95.4
hRF_RLR	96.0	92.3
hRF_SVM	97.1	90.5
hRF_DT	95.4	93.1
hRF_NB	96.3	90.2
hSVM_LR	85.9	94.8
hSVM_RF	88.0	94.2
hSVM_DT	92.5	93.4
hSVM_NB	83.0	91.7
hNB_LR	85.9	95.4
hNB_RF	87.9	96.0
hNB_DT	95.9	90.9
hNB_SVM	83.0	91.6

To illustrate the diagnostic ability of our classifier, we create a graphical plot called receiver operating characteristic (ROC) curve, showing the graphical plot while varying the discrimination threshold. The ROC curve is a plot of the true positive rate (TPR) on the y-axis versus the False Positive Rate (FPR) on the x-axis for every possible classification threshold.

In machine learning, the true-positive rate, also known as sensitivity or recall answers the question how does the classifier predict positive when the actual classification is positive (i.e. not healthy). On the other hand, the false-positive rate, which is also known as the fall-out or probability of false alarm answers the question how does the classifier incorrectly predict positive when the actual classification is negative (i.e. healthy).

**Fig. 9:**
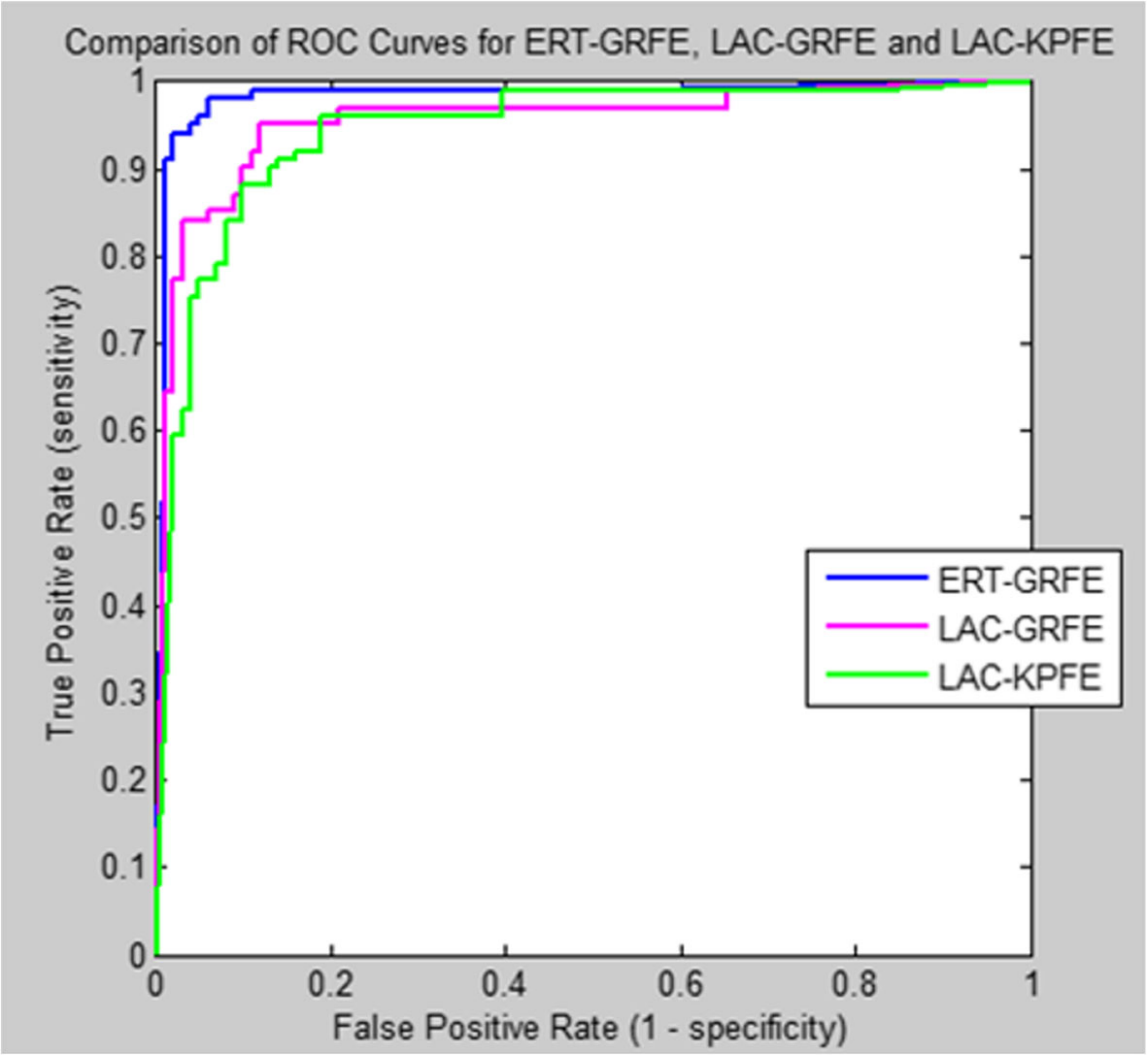
Comparison of the ROC curve of our classifiers using different feature extraction methods (ERT-GRFE, LAC-GRFE and LAC-KPFE) for Healthy and Not Healthy classification

**Fig. 10:**
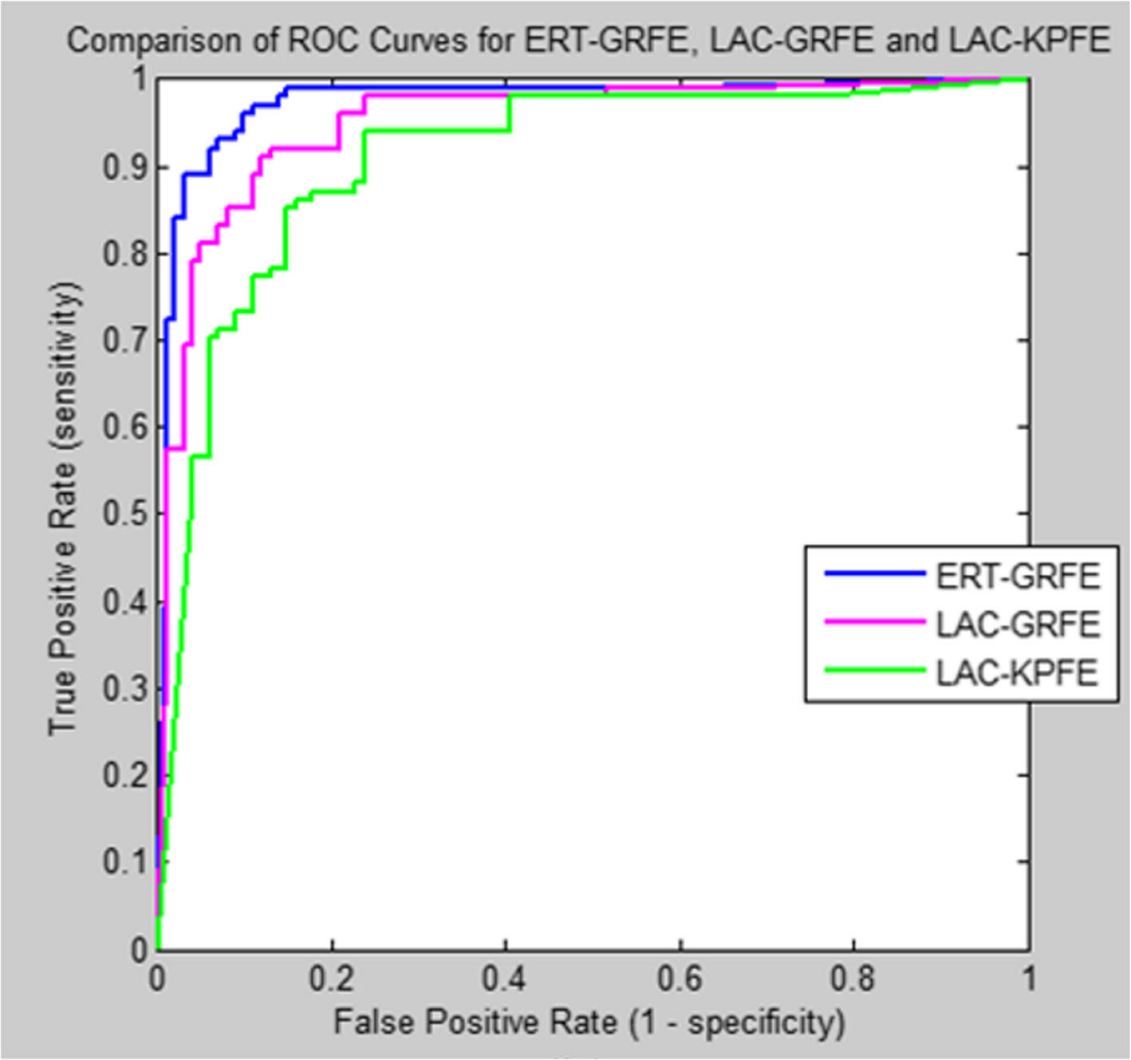
Comparison of the ROC curve of our classifiers using different feature extraction methods (ERT-GRFE, LAC-GRFE and LAC-KPFE)for Facial Palsy classification

The ROC curve is therefore the sensitivity as a function of fall-out. Figures 9 and 10 present the comparison of the area under ROC curve (AUC) of our hybrid hDT_RLR classifier for healthy and unhealthy discrimination and for FP type classification (i.e. central or peripheral), respectively, using three different feature extraction methods: (a)Localized Active Contour-based method for key points extraction (LAC-KPFE));(b)Localized Active Contour-based method for geometric and region features extraction (LAC-GRFE); and (c)Ensemble of Regression Tree-based method for geometric and region features extraction (ERT-GRFE).

Our proposed approach ERT-GRFE with hybrid classifier hDT_RLR achieves better performance in AUC of 2.7%-4.9% higher than other two methods in discriminating healthy from unhealthy (i.e. with paralysis) subjects as shown in Fig. 8. Similarly, for the palsy type classification: central palsy (CP) and peripheral palsy (PP), ERT-GRFE plus hDT_RLR hybrid classifier outperformed the two (2) feature extraction methods LAC-GRFE and LAC-KPFE used in the experiments with at least 2.5%-7.7% as in Fig. 9. Experiments reveal that our method yields more stable results.

Tables 3 and 4 present a comparison of the performance of the three methods for discriminating healthy from unhealthy subjects; and for classifying facial palsy type, respectively. Each approach differs according to the features applied; and the corresponding methods used for extracting such features, which include: (a)Localized Active Contour-based method for key points feature extraction (LAC-KPFE));(b)Localized Active Contour-based method for geometric and region-based features (LAC-GRFE); and (c)Ensemble of Regression Tree-based method for geometric and region-based feature extraction (ERT-GRFE).

**Table 3:** Comparison of the performance of the three methods for healthy and unhealthy discrimination

	LAC-based	LAC-based	ERT-based GRFE
	KPFE	GRFE	(our approach)
Sensitivity	89.12%	95.01%	98.12%
Specificity	90.01%	88.12%	94.06%
AUC	93.40%	95.56%	98.34%

**Table 4:** Comparison of the performance of the three methods for facial palsy classification

	LAC-based	LAC-based	ERT-based GRFE
	KPFE	GRFE	(our approach)
Sensitivity	85.15%	91.09%	97.48%
Specificity	85.12%	88.10%	94.91%
AUC	89.81%	95.01%	97.48%

Table 3 shows that in discriminating healthy from unhealthy subjects, our proposed approach outperforms other methods that use key or salient points-based features using LAC model, in terms of sensitivity and specificity with the improvement of 9% and 4.1%, respectively. Similarly, experiments show that our approach outperforms the previous method [4] that used geometric and region-based features (GRFE) using LAC model in terms of sensitivity and specificity with an improvement of 3.1% AND 5.9%, respectively. On the other hand, Table 4 reveals that for central and peripheral palsy classification, our proposed ERT-based GFRE is way better than the previous approach that solely used key points-based features with an improvement of around 12% and 9%, of the sensitivity and specificity performance measure, respectively. Furthermore, experiments reveal that our ERT-based GFRE proposed approach yields better performance, particularly in sensitivity and specificity with an improvement of 6.4% and 6.8%, respectively. Thus, our proposed approach is superior among other three methods.

## Discussion

Empirical results [15] reveal that ensemble of regression trees (ERT) model does have an appealing quality that it performs shape invariant feature selection while minimizing the same loss function during training and test time, which significantly lessens time complexity of extracting features. True enough, our experiments reveal that ERT-based method for geometric and region features extraction (ERT-GRFE) works well. Furthermore, our proposed approach to combine iris segmentation and the ERT-based key point detection for feature extraction provides a better discrimination of central and peripheral palsy most especially in ‘raising of eyebrows’ and ‘screwing of nose’ movements. It shows changes of the structure on edges of the eye, i.e., the significant difference between the normal side and the palsy side for some facial movements (e.g. eyebrow lifting, nose screwing, and showing of teeth). Also, features based on the combination of iris and key points by utilizing ensemble of regression tree technique can model the typical changes in the eye region. A closer look at the performance of our classifier, as shown in Tables 3 and 4 reveal interesting statistics in terms of the specific abilities of the three methods. Our method proves to have significant contribution in discriminating central from peripheral palsy patients and healthy from facial palsy subjects. The combination of iris segmentation and ERT-based key point approach is more suitable for this operation.

## Conclusion

In this study, we present a novel approach to address FP classification problem in facial images. Salient points and iris detection based on ensemble of regression trees are employed to extract the key features. Regularized Logistic Regression (RLR) plus Classification Tree (CT) classifier provides an efficient quantitative assessment of the facial paralysis. Our facial palsy type classifier provides a tool essential to physicians for deciding the medical treatment scheme to begin the patient’s rehabilitation process. Furthermore, our approach has several merits that are very essential to real application:

- geometric and iris region features based on ensemble of regression trees and optimized Daugman’s theory (using parabolic function, i.e. 2^*nd*^ degree polynomial), respectively, allows efficient identification of the asymmetry of the human face, which reveals the significant difference between the normal and afflicted side, which localized active contour model fail to track especially for peculiar images (e.g. wrinkles, excessive mustache, occlusions, etc.);
- ERT model does have have a very appealing quality that reduces errors in each iteration, which can be very useful in extracting boundaries of the facial features from the background; and
- ERT model does not require proper or perfect identification of initial evolving curves to ensure accurate facial feature extraction.
- our method significantly lessens the time complexity of extracting features, without sacrificing the level of accuracy making it more suitable for the real application.

## Abbreviations

1D: 1 Dimensional
AUC: area under ROC curve
CP: Central palsy
CT: Classification tree
DT: Decision Tree
ERT: Ensemble of regression trees
ERT-GRFE: Ensemble of Regression Tree-based method for geometric and region features extraction
FP: Facial Paralysis
FPR: False Positive Rate
HOG: Histogram of Oriented Gradients
hDT_RLR: hybrid Decision tree and Regularized Logistic Regression
IC: Inner Canthus
IO: Infra Orbital
IRB: Institution Review Board
LAC: Localized Active Contour
LAC-GRFE: Localized Active Contour-based method for geometric and region features extraction
LAC-KPFE: Localized Active Contour-based method for key points extraction
MA: Mouth Angle
ML: Machine learning
NB: Naive bayes
NT: Nose tip
OC: Outer Canthus
PP: Peripheral palsy
RF: Random Forest
RLR: Regularized Logistic Regression
ROC: Receiver operating characteristic curve
ROI: Region of interest
SO: Supra-orbital
SVM: Support Vector Machine
TPR: True positive rate
UE: Upper Eyelids
